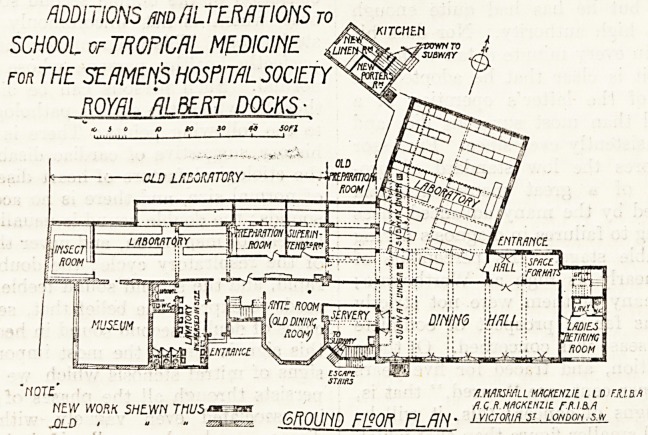# The London School of Tropical Medicine

**Published:** 1913-09-20

**Authors:** 


### THE LONDON SCHOOL OF TROPICAL MEDICINE.

### Its Latest Developments in Building and Teaching.

The new buildings which have been in course of construction during the last year, and which have been rendered possible by the generous response which has been made to Mr. Chamberlain's appeal, are now practically complete, and a visitor to the Albert Dock will be repaid by seeing one of the most complete schools of medicine in the kingdom.

The old laboratories with their attendant museum, library, etc., were spacious, and gave accommodation for about ninety students, but this accommodation is now greatly increased. The new laboratory, which is a single-storey building with a weaver roof, will hold seventy students. The older laboratories have been sub-divided, and now give accommodation for a room for the director, a preparation room, an insectarium, and a special laboratory, in which will be carried on the new course in tropical sanitation and hygiene, of which more anon.

The equipment throughout is of the very latest design. Teak-topped benches, with a sink and lights, for every two students. There is no one seat in the large laboratory better than another, every one deriving a clear view of the heavens in the mirror of his microscope.

A new feature is the provision of special accommodation for women students, of whom there are an ever-increasing number attending the school, suitable retiring rooms for writing, etc., being provided.

New incubating centres have been installed, and the general provision for a supply of material for the large class of sixty which assembles for the ordinary course every session is readily at hand.

The only parts of the schools that have not been altered are the special departments for entomology, helminthology, and protozoology. These remain as before, giving facilities for about forty advanced students.

The new course in tropical sanitation and hygiene, which will be inaugurated during the October session, will extend over a period of two months of regular laboratory tuition with demonstrations, and in addition to this there will be an extra month of study for those who desire to be signed up for three months. The new course, together with the ordinary course, will then cover six months, the period required to enable students to sit for the diploma in the diseases and hygiene of the tropics (D.T.M. Eng.) granted by the Conjoint Board. Among the subjects included in the course of tropical sanitation and hygiene are:—Bacteriology, chemistry, hygiene, surveying, and entomology. This will necessitate the appointment of a bacteriologist to devote the whole of his time to the course. There will also be a specially appointed chemist and a surveyor, while hygiene and entomology will be dealt with by some of the teachers in the ordinary course.

The carrying out of the proposals for a course in tropical sanitation and hygiene have been brought about by the Report of the Board of Education, and the cost will be defrayed in part by the fees of the students, and in part by the endowment created by Mr. Chamberlain's fund.

The residential accommodation, or the hostel, as the accommodation for resident students is so frequently termed nowadays, has been greatly extended, and the rooms which have hitherto accommodated about ten students have been greatly added to. Up to the present it has not been possible to provide accommodation for anything like the number of students who desire to live in the school. There will now be room for twenty-five students, and in connection with this there has been erected a large dining room, the old dining room being converted into an ante-room. The library has been enlarged, and the general administrative premises have been extended in every direction.

## Figures and Tables

**Figure f1:**
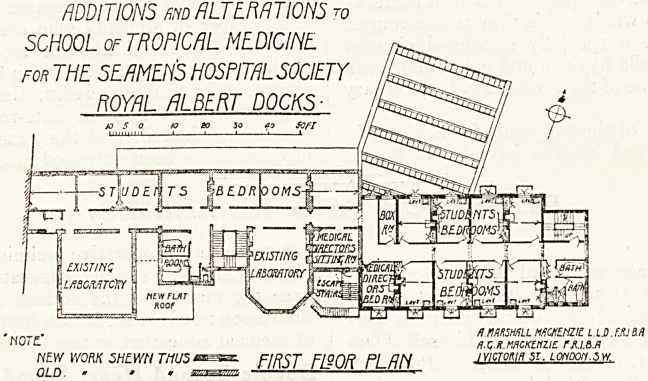


**Figure f2:**